# Synergy of endothelial and neural progenitor cells from adipose-derived stem cells to preserve neurovascular structures in rat hypoxic-ischemic brain injury

**DOI:** 10.1038/srep14985

**Published:** 2015-10-08

**Authors:** Yuan-Yu Hsueh, Ya-Ju Chang, Chia-Wei Huang, Fitri Handayani, Yi-Lun Chiang, Shih-Chen Fan, Chien-Jung Ho, Yu-Min Kuo, Shang-Hsun Yang, Yuh-Ling Chen, Sheng-Che Lin, Chao-Ching Huang, Chia-Ching Wu

**Affiliations:** 1 Division of Plastic Surgery, National Cheng Kung University Hospital, North District, Tainan City, Taiwan; 2 Institute of Clinical Medicine, National Cheng Kung University, North District, Tainan City, Taiwan; 3 Department of Cell Biology and Anatomy, National Cheng Kung University, North District, Tainan City, Taiwan; 4 Institute of Basic Medical Sciences, National Cheng Kung University, North District, Tainan City, Taiwan; 5 Department of Occupational Therapy, I-Shou University, Kaohsiung City, Taiwan; 6 Department of Physiology, National Cheng Kung University, North District, Tainan City, Taiwan; 7 Department of Pediatrics, Taipei Medical University, Xinyi District, Taipei City, Taiwan; 8 Institute of Oral Medicine, National Cheng Kung University, North District, Tainan City, Taiwan; 9 Department of Pediatrics, Wan-fan Hospital, College of Medicine, Taipei Medical University, Xinyi District, Taipei City, Taiwan; 10 Department of Biomedical Engineering, National Cheng Kung University, North District, Tainan City, Taiwan; 11 Medical Device Innovation Center, National Cheng Kung University, North District, Tainan City, Taiwan

## Abstract

Perinatal cerebral hypoxic-ischemic (HI) injury damages the architecture of neurovascular units (NVUs) and results in neurological disorders. Here, we differentiated adipose-derived stem cells (ASCs) toward the progenitor of endothelial progenitor cells (EPCs) and neural precursor cells (NPCs) via microenvironmental induction and investigated the protective effect by transplanting ASCs, EPCs, NPCs, or a combination of EPCs and NPCs (E+N) into neonatal HI injured rat pups. The E+N combination produced significant reduction in brain damage and cell apoptosis and the most comprehensive restoration in NVUs regarding neuron number, normal astrocytes, and vessel density. Improvements in cognitive and motor functions were also achieved in injured rats with E+N therapy. Synergistic interactions to facilitate transmigration under *in vitro* hypoxic microenvironment were discovered with involvement of the neuropilin-1 (NRP1) signal in EPCs and the C-X-C chemokine receptor 4 (CXCR4) and fibroblast growth factor receptor 1 (FGFR1) signals in NPCs. Therefore, ASCs exhibit great potential for cell sources in endothelial and neural lineages to prevent brain from HI damage.

Injuries in the central nervous system (CNS), such as stroke or cerebral vascular lesions, are devastating with permanent neuronal damage and lifelong functional loss. During childbirth, perinatal cerebral hypoxic and ischemic (HI) injury due to intrapartum asphyxia is a major cause of neonatal morbidity and mortality^1^. Birth asphyxia causes global ischemia of the brain, and approximately half of the survivors have long-term pathological outcomes, including seizures and neurological deficits^2^.

The neurovascular unit (NVU) is a dynamic structure consisting of endothelial cells, basal lamina, pericytes, astrocytic end-foot processes, and neurons that determines the integrity of inter-endothelial tight junctions and the interaction among astrocytes, endothelial cells, and neurons^3^. After cerebral HI injury, the architecture of the NVU is disordered, and the permeability of the blood–brain barrier is increased, which further damages the neurological structures. Conventional therapies, such as up-regulation of endothelial nitric oxide synthase and application of L-arginine and statins can alleviate symptoms only partially, and the patients remain in a state of sustained disability^4^,^5^.

Transplantation of endothelial progenitor cells (EPCs) is a cell-based therapy aimed at revascularizing the ischemic tissue^6^ or site of traumatic brain injury^7^. However, the scarcity of EPCs and the difficulty in isolating these cells led researchers to identify alternative sources, such as embryonic stem cells (ESCs)^8^, bone marrow mesenchymal stem cells (MSCs)^7^,^9^, and fetal umbilical cord blood^10^. Yet, the considerations of tumorigenicity and limited resources still exist with these sources. On the other hand, the CNS also shows poor self-regeneration ability after injury and requires transplantation of neural stem cells (NSCs) and/or neural precursor cells (NPCs) to repair the nervous system for functional recovery^11^. NSCs and/or NPCs may be obtained from ESCs^12^ or induced pluripotent stem cells^13^, and NSCs may be directly harvested from fetal or adult nervous system tissue^14^ or trans-differentiated MSCs^15^. However, the source of fetal brain tissue is limited, and the recipient patients require immunosuppressive treatment after cell therapy. The genetic instability and risk of teratoma formation with ESCs and induced pluripotent stem cells also prohibit the application of these cells in clinical trials^16^.

Adipose-derived stem cells (ASCs), isolated from adipose tissue, belong to the family of MSCs and can be differentiated into multiple lineages via chemical induction factors^17^. ASCs share common genetic signals with bone marrow MSCs and have additional advantages, such as abundant quantities, minimally invasive procedures for harvest, and autologous origins that will not require immunosuppression in future therapies^18^. The conditioned medium of ASCs protects neonatal rats against HI-induced brain damage^19^. ASCs express endothelial and neural progenitor markers after differentiation, which can improve postnatal neovascularization^20^. Our recent studies also demonstrate sphere formation with neural-specific gene and protein expression by seeding the ASCs on chitosan-coated surfaces, and significant improvement in functional recovery following sciatic nerve regeneration^21^,^22^. In addition, endothelial differentiation can be induced in human placenta-derived multipotent cells (PDMCs) with synergistic simulation using endothelial growth medium (EGM) and subsequent exposure to fluid laminar shear stress (LSS)^23^. The differentiated PDMCs show increased gene and protein expression for endothelial markers, such as von Willebrand Factor (vWF) and platelet-endothelial cell adhesion molecule-1 (PECAM-1), and demonstrate endothelial functions such as uptake of acetylated low-density lipoproteins (acLDL) and formation of tube-like structures on Matrigel. Therefore, the microenvironmental cues may facilitate the differentiation ability of ASCs toward endothelial or neuronal lineages to become sources of EPCs and NPCs. The current study aims to establish therapeutic cells derived from ASCs and use them in neonatal animals with brain HI injury to evaluate the therapeutic effectiveness and to understand the protective mechanism of specified cell therapy.

## Results

### Inducing ASCs to differentiate into EPCs and NPCs

**Figure 1:**
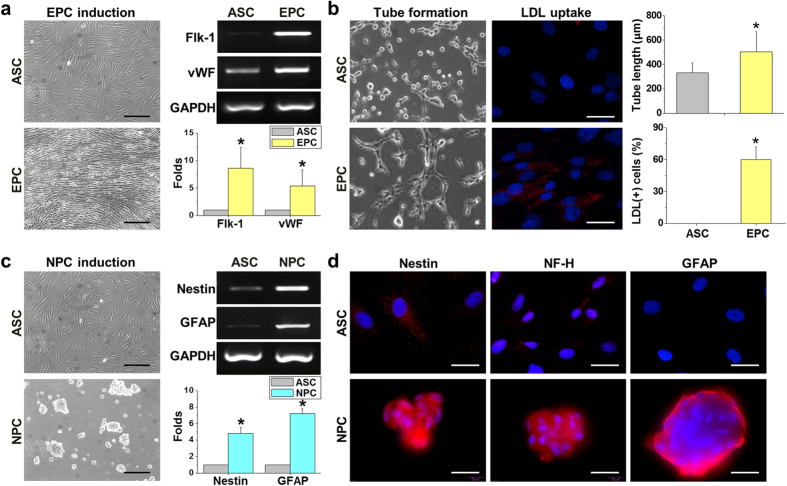
After endothelial growth medium treatment and laminar shear stress stimulation, the EPCs differentiated from human ASCs were aligned in parallel to the direction of fluid flow and showed an increase in the endothelial markers Flk-1 and vWF (n = 15). (**a**) Endothelial functions exhibited increased tube-like structures on Matrigel (n = 10) and uptake of DiI-labeled acLDL (n = 10). (**b**) Neural differentiation was induced by forming free-floating spheres on the chitosan-coated surface. Spheres expressed the specific differentiation markers nestin and GFAP for neural lineages (n = 13). (**c**) Immunofluorescent staining showed an increase in nestin, NF-H, and GFAP in the spheres (n = 10). (**d**) Scale bar in phase image: 200 μm. Scale bar in fluorescent image: 50 μm. Data are represented as mean ± SD. *p < 0.05 compared to undifferentiated ASCs.

Human ASCs were induced to differentiate into EPCs by pretreating them with EGM for 3 days and then subjecting them to LSS for 24 hrs. The undifferentiated ASCs showed mesenchymal spindle-like morphology. After EPC differentiation, the cells were able to sense the direction of fluid shear stress as observed with parallel alignment of the cells (Fig. 1a). EPC induction significantly increased the expression of endothelial markers, such as Flk-1 (vascular endothelial growth factor receptor 2 (VEGFR2) and vWF. Contrary from previous results in placenta multi-potent cells^23^, the ASCs exhibited minor expression of endogenous vWF before differentiation. The formation of tube-like structures on Matrigel and the ability to endocytose DiI-labeled acLDL confirmed proper endothelial functions after EPC induction (Fig. 1b).

NPCs were derived by seeding human ASCs on chitosan-coated surfaces where they formed sphere-like structures after culturing for 3 days (Fig. 1c). The undifferentiated ASCs expressed low levels of the endogenous neurological markers such as nestin and glial fibrillary acidic protein (GFAP) genes. After sphere formation, significant induction of both nestin and GFAP gene expression indicated these human ASCs were directed towards neural lineages (Fig. 1c). The induction of NPCs was further confirmed by immunofluorescent staining showing positive expression of neural-specific proteins nestin, neurofilament heavy chain (NF-H), and GFAP (Fig. 1d).

To demonstrate human ASCs have differentiation and therapeutic potential that may benefit future clinical application, ASCs were primary isolated from the raw lipoaspirates of healthy donors. The induction of EPCs and NPCs from ASCs was also assessed by isolating ASCs from 6-weeks old rats. Similar to ASCs from human tissue, the rat ASCs showed; increased endothelial gene expression (Fig. S1A), the ability to form tube-like structures (Fig. S1B), and the uptake of acLDL (Fig. S1C) after EPC induction. The rat ASCs also demonstrated NPC induction after seeding on chitosan-coated surfaces (Fig. S1D). These results indicate that ASCs from both humans and rats have the potential to differentiate into EPCs and NPCs, and act as alternative cell sources.

### Protecting the brain from infarction after HI injury

**Figure 2:**
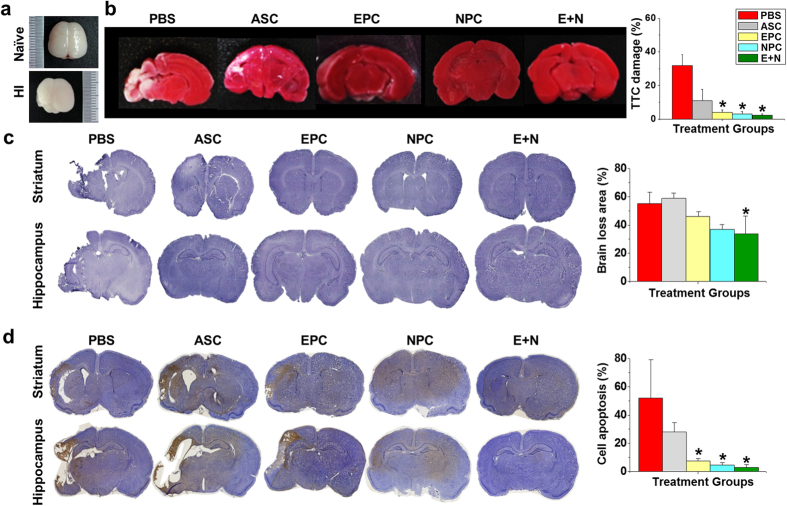
Hypoxic-ischemic (HI) injury was created in neonatal rats on postnatal day 7, and the brain was harvested 7 days after injury (n = 4). (**a**) Prevention of brain tissue loss was measured by TTC staining after different specified cell therapies (n = 5). (**b**) Nissl staining revealed that the application of EPCs or NPCs alone minimally prevented brain loss compared to injection of the original ASCs (n = 5). (**c**) The combination treatment of EPCs and NPCs (E+N) further improved the therapeutic effect in live neurons. Cell apoptosis (brown color) in HI-injured brains was observed with TUNEL staining (n = 5). (**d**) The E+N combined treatment showed the best outcome in protecting the brain cells from death after HI injury. *p < 0.05 compared to PBS-injected rats.

After we confirmed the plasticity of ASCs for endothelial and neural differentiation, the HI encephalopathy model was created in postnatal day 7 rats as described in a previous study^24^. The HI-injured rat pups were divided into five groups that received PBS injection or transplantations of ASCs, EPCs, NPCs, or a combination of EPCs and NPCs (E+N). To evaluate the therapeutic effect of different cell types, the brains were harvested after treatment for 7 days (Fig. 2a). The 2,3,5-triphenyltetrazolium chloride (TTC) staining demonstrated severe brain damage after HI injury (Fig. 2b, white color in PBS brain section). Transplantation of E+N combination showed significant preservation effects compared to the brain damage of animals that received the sham PBS injection (Fig. 2b). The protection from brain damage was further verified by Nissl staining of live neurons and demonstrated a decrease in brain damage after application of EPCs, NPCs, and the combination of E+N (Fig. 2c). Similar to the multiple progenitor cell types derived from human ASCs, the ASCs isolated from rats and their progenitor cells also demonstrated protection of the brain from tissue damage after HI injury (Fig. S2). The DNA fragments in dead cells after HI injury were assessed with terminal deoxynucleotidyl transferase dUTP nick end labeling (TUNEL) staining of brain sections (Fig. 2d.). Large numbers of brown apoptotic cells (TUNEL-positive cells) were observed in the injured hemisphere after PBS or ASC treatments. Single transplantation of EPCs or NPCs had a partial therapeutic effect in prevention of cell apoptosis. However, the E+N combination showed the most significant outcome to protect brain cells from HI-induced cell death.

### Preserving the NVU structure with specific cell treatments

**Figure 3:**
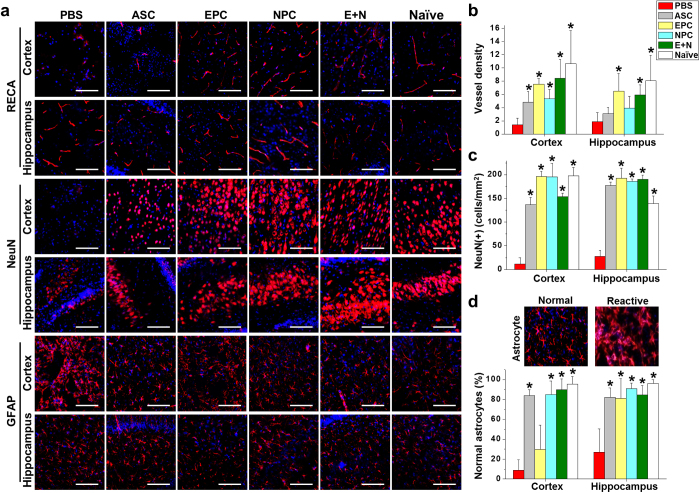
Improvements in neurovascular structures in HI-injured brain were evaluated with specific staining for vessels (RECA, n = 8), neurons (NeuN, n = 7), and astrocytes (GFAP, n = 7) in the cortex and hippocampus. (**a**) The microvascular structure of the injured hemisphere of HI brains was measured by vessel density in the RECA-stained images after different specified cell therapies. (**b**) Cortical vessel density was increased after the transplantation of ASCs, EPCs, NPCs, or E+N combined therapy. However, the vessel density in the hippocampus was increased when treated with EPCs or the E+N combination. Increases in NeuN (+) cells were observed in the cortex and hippocampus of rats treated with ASCs, EPCs, NPCs, or the E+N combination. (**c**) After HI injury, staining with GFAP showed reactive astrocyte morphology without cell therapy. (**d**) Injection of ASCs, NPCs, or the E+N combination inhibited astrocyte activation, but this was not observed with EPC treatment. Scale bar: 100 μm. *p < 0.05 compared to PBS-injected rats.

The NVU structures in HI-injured brain were assessed with immunofluorescent staining of frozen sections with specific antibodies against endothelial cells (RECA) for vessels, neuron-specific nuclear protein (NeuN) for neurons, and GFAP for astrocytes (Fig. 3a). The preservation of brain integrity was illustrated by the NVU structures in the cortex and hippocampus. In RECA staining images, the vascular structure was severely disrupted in the cortex with a lower level of vessel damage seen in the hippocampus of HI-injured rat pups without cell therapy (sham PBS injection). The vessel density was restored to normal brain levels (naïve) after EPC or E+N combination therapies in both cortex and hippocampus (Fig. 3b). The transplantation of NPC only showed a less therapeutic effect in vascular structure and density, especially in the hippocampus (Fig. 3b). NeuN-positive cells were significantly decreased in both the cortex and hippocampus of HI-injured brains (Fig. 3a, NeuN staining). Transplantation of ASCs, EPCs, NPCs, or the E+N combination significantly increased the number of NeuN-positive cells in both the cortex and hippocampus compared to the sham PBS injection (Fig. 3c). GFAP staining revealed reactive astrocyte morphology in the cortex after HI injury (Fig. 3a, GFAP staining). A significant increase of gliosis was observed to indicate the pathological remodeling without cell therapy. Injection of ASCs, NPCs, or the E+N combination reduced the number of reactive astrocytes and increased the number of normal astrocytes (Fig. 3d). However, single transplantation of EPCs did not have a therapeutic effect in reducing reactive astrocytes in the cortex (Fig. 3d). NVU structures in other brain areas, such as the striatum, also showed similar protective effects as seen in the cortex and hippocampus (Fig. S3). These results demonstrated that EPC and NPC play different roles in restoring the vessel and glia functions, respectively. The E+N grouped these beneficial effects in preserving the NVU structure after HI injury.

### Cell engraftment and contributions in neurovascular structure

**Figure 4:**
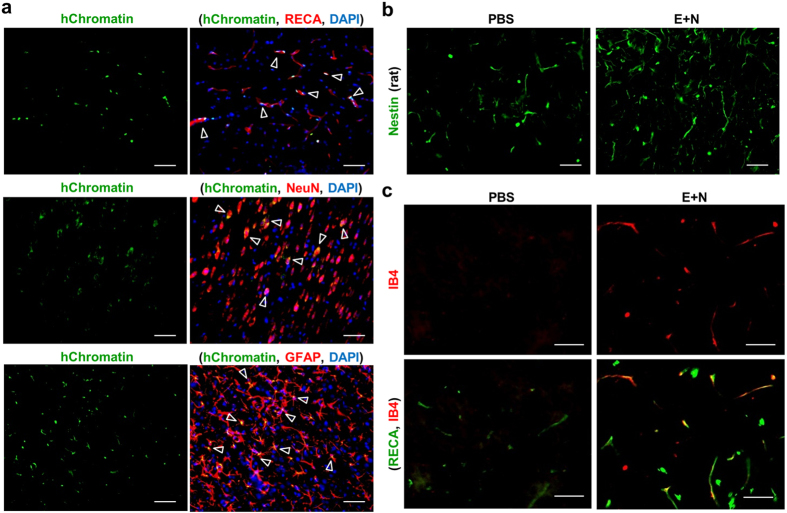
After transplantation of EPCs and NPCs derived from human ASCs, the engraftment of differentiated cells was identified with double staining (arrowheads) of human chromatin (hChromatin, green) and by specific antibodies against RECA (n = 5), NeuN (n = 5), and GFAP (n = 5) (red). (**a**) The E+N combined therapy promoted endogenous neurogenesis as indicated by increased nestin staining specific for rat neural stem cells, but not human nestin. (**b**) The combination of E+N also increased angiogenesis as demonstrated by increased isolectin IB4 staining (n = 5). (**c**) Scale bar: 50 μm.

To visualize the transplanted cells, they were labeled with green fluorescent protein (GFP) using adenovirus transduction (ad-GFP) 48 hrs before the injection and achieved high expression efficiency (higher than 80%) in both human and rat cells (Fig. S4). The differentiation of ASCs into EPCs or NPCs by environmental induction did not alter the expression of GFP in these progenitor cells. In addition, the engrafted cells were derived from human ASCs, which can be identified by double staining of human-specific chromatin (hChromatin) plus NeuN, GFAP, and RECA in the cortex after injection of the combination of EPCs and NPCs (Fig. 4a). The double-positive, yellow cells indicated the engrafted cells (green color) contributed to a different composition of NVU structures (red color) after combined treatment. Because other NVU structures may contribute by mediating the neurogenesis and angiogenesis, we further examined the beneficial effects of increased endogenous progenitor cells. A species specific antibody directed against rat nestin, but does not react with human nestin, demonstrated that the transplantation of the E+N combination promoted the differentiation of endogenous neural progenitor cells (Fig. 4b). The combined therapy also promoted angiogenesis as identified by isolectin IB4 conjugate antibody (Fig. 4c).

### Recovery of motor and memory function

**Figure 5:**
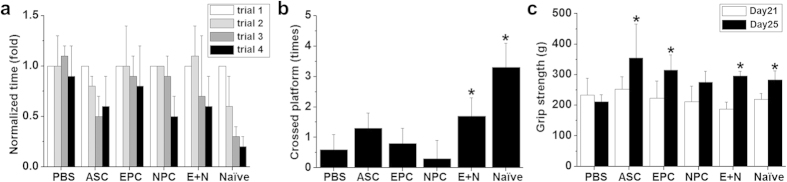
Cognitive function was evaluated using the Morris water maze after animals received different cell transplantations. The HI-injured rats showed impairment in learning ability, which was improved by all cell therapies (n = 9). (**a**) No significant differences in normalized time were found among the various treatments in learning ability. In memory function, the numbers of platform crosses were significantly increased in rats that received the E+N combined treatment (n = 9). (**b**) Motor function was measured by grip strength and showed improvements in rats with ASCs, EPCs, or the E+N combined treatment (n = 9). (**c**) *p < 0.05 compared to PBS-injected rats.

The Morris water navigation task was performed to test the brain function of rat pups 14 days after receiving cell transplantation (postnatal day 21). The normal rats (naïve) were able to memorize the landmark in the water maze, and showed incrementally decreased latency time to reach the platform during different trials (Fig. 5a). When we removed the platform, the rats showed an increased number of times crossing the previous location of the platform, indicating that they had memorized the location (Fig. 5b). Impairments in both learning and memory were observed in the rats after HI injury (PBS injection). Transplantation of different types of cells seemed to improve the learning ability, but results were not statistically significant different due to overlapping standard deviations (Fig. 5a). In contrast, significantly increased numbers of platform location crosses in the Morris water maze test revealed that rats with E+N combined transplantation had better memory performance than rats treated with the sham PBS (Fig. 5b). The immunofluorescent staining of NeuN in cortex after the memory test (postnatal day 25) demonstrated the increase of NeuN-positive cells with transplantation of EPCs, NPCs, and E+N combination (Fig. S5). The maximum grip force, demonstrates impairment in motor function in rats after HI injury, was restored by transplanting ASCs, EPCs, or the E+N combination (Fig. 5c).

The therapeutic effects of cell-based treatment by transplanting different cell types derived from ASCs are summarized in Table 1. The treatment of injecting ASCs showed minor therapeutic effects compared to injured rats without treatment (sham PBS injection). Either endothelial or neural differentiation to induce ASCs toward EPCs or NPCs had superior therapeutic outcomes with specific functional roles to preserve the NVU structures. However, partial limitations were observed when transplanting single types of progenitor cells for reducing reactive astrocytes using EPC treatment and for increasing vessel density using NPC treatment. The combination of EPCs and NPCs showed the most promising outcomes to prevent infarction (TTC), preserve living neurons (Nissl), prevent cell death (TUNEL), increase NeuN (+) neurons, increase normal astrocytes, improve vessel structure, and improve functions in memory and motor performance (Table 1).

### Synergistic interactions between EPCs and NPCs

**Figure 6:**
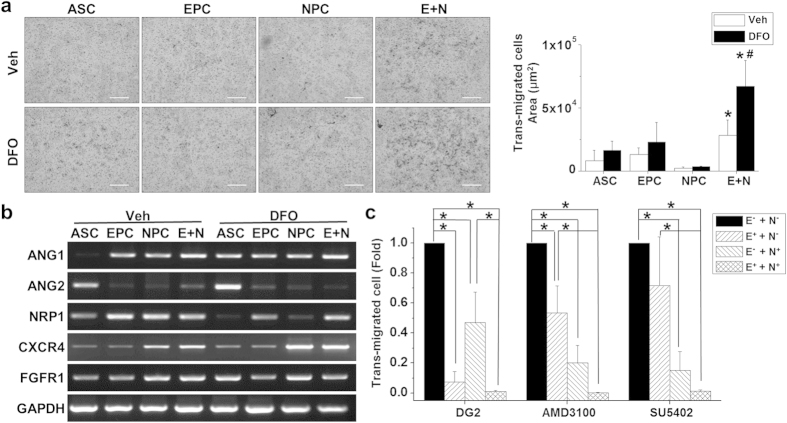
The combination of EPCs and NPCs increased transmigration in a Boyden chamber under normoxic conditions, and the application of the hypoxia mimetic reagent DFO further promoted the mobility in the E+N group (n = 5). (**a**) Different types of cells with or without DFO treatment were harvested to assess the potential gene expression in angiogenesis and neurogenesis signaling pathways for ANG1, ANG2, NRP1, CXCR4, and FGFR1 (n = 3). (**b**) Specific inhibitions of angiogenic (NRP1 inhibiting peptide, DG2) and neurogenic (AMD3100, a CXCR4 inhibitor and SU5402, an FGFR1 inhibitor) signaling were applied to EPCs or NPCs (n = 5). (**c**) Synergistic interactions were originated from EPCs via NRP1 signaling and from NPCs by CXCR4 and FGFR1 signaling pathways. Scale bar: 200 μm. *p < 0.05 compared to undifferentiated ASCs under normoxia. ^#^p < 0.05 compared to undifferentiated ASCs under hypoxia.

To further illustrate the beneficial combination of EPCs and NPCs, cell migration was investigated using both the wound closure and transmigration assays under normoxia and hypoxia conditions. By seeding equal numbers of cells in Boyden chambers, the E+N combination showed an increase in cell motility under hypoxic conditions when treated with the hypoxia mimetic desferrioxamine (DFO) (Fig. 6a). This suggests that some cell-cell interactions or synergistic effects were triggered when transplanting the EPCs and NPCs together into the physiologically hypoxic-injured rats. The synergistic benefit of EPCs and NPCs in transmigration was also confirmed in the differentiated cells from rat ASCs (Fig. S6). The underlying interactive mechanism for potential benefits in angiogenesis and neurogenesis from EPCs and NPCs was probed by examining gene expression for angiopoietin-1 (ANG1), angiopoietin-2 (ANG2), neuropilin-1 (NRP1), C-X-C chemokine receptor type 4 (CXCR4), and fibroblast growth factor receptor 1 (FGFR1) (Fig. 6b). The undifferentiated ASCs expressed ANG2, but not ANG1. Both endothelial and neural differentiation increased ANG1 expression in EPCs and NPCs. Additionally, the increased induction of ANG1 expression in the E+N combination suggested a synergistic effect between EPCs and NPCs. DFO treatment increased both ANG1 and ANG2 gene expression in undifferentiated ASCs, but did not alter ANG1 expression in differentiated cells or cell combinations. NRP1, another angiogenesis-related signaling pathway, was also increased in differentiated EPCs and NPCs. Interestingly, DFO treatment reduced NRP1 expression in ASCs and NPCs, but the NRP1 levels in EPCs and the E+N combination still remained the same compared to normoxic conditions. This indicates that the resistance of NRP1 expression in EPCs and the combined E+N therapy may play an important role in maintaining angiogenesis function under hypoxic conditions. The gene expression of CXCR4 was increased when differentiating ASCs into NPCs. The combination of NPCs with EPCs did not alter CXCR4 expression. DFO treatment further increased CXCR4 gene expression in both NPCs and the E+N combination. The gene expression of FGFR1 was increased in the NPC and combined E+N groups under normoxic conditions. DFO treatment increased FGFR1 expression in ASCs, but slightly decreased its expression in the E+N combination. In summary, these results indicate that EPCs and NPCs may interact through multiple signaling pathways. We further explored the possible interaction mechanism by adding specific inhibitors to each type of progenitor cells before the combination of EPCs and NPCs under hypoxic conditions (Fig. 6c). Blockage of NRP1 signals by specific NRP1 peptides (DG2) in EPCs decreased the synergistic effect of the E+N combination under hypoxia. The inhibition of CXCR4 signals by pretreating the NPCs with AMD3100 (a specific CXCR4 antagonist) for 1 hr abolished the induction of transmigration in the E+N combination. The FGFR1 inhibitor (SU5402) also inhibited transmigration in NPCs (Fig. 6c).

## Discussion

Stem cell therapies are useful, promising treatments for tissue regeneration and repair, especially for degenerating, ischemic, and inflammatory tissues. Both ASCs and MSCs were used to reverse the brain damage, but ASCs are more abundant and easier to harvest than bone marrow MSCs. The therapeutic application of EPCs, which persist in the bone marrow and circulate in humans, has been tested in many cardiovascular trials^25^. EPCs express endothelial markers, such as CD34+/CD133+/Flk-1+or CD31, and can migrate from the peripheral circulation to the injured site to form new vessels or differentiate into mature endothelial cells in animal models of ischemia^26^. Other endothelial cells, such as human umbilical vein endothelial cells, also show the ability to repair neonatal HI brain injury^24^. The current study provides a convenient method to obtain EPCs from ASCs (Fig. 1) and confirms their therapeutic effects in preventing HI brain injury in rats (Fig. 2). The induction of EPCs by EGM was also established in bone marrow mesenchymal stem cells (BMMSCs) and used for treatment of traumatic brain injury in rats^7^. Our findings suggest that the combined usage of EGM and LSS synergistically promotes endothelial differentiation of ASCs. The gene expression patterns were slightly different from PDMCs^23^ because pretreatment with EGM already induced minor expressions of Flk-1 and vWF in ASCs (Fig. 1a). However, subsequent LSS treatment still promoted a significant increase in endothelial cell differentiation as indicated by endothelial gene expression and function. Interestingly, Flt-1 (VEGFR1) was increased in differentiated PDMCs, but was not altered by either EGM or LSS in ASCs. These results suggest that endogenous variations in adult stem cell characteristics exist between ASCs and PDMCs, but both can respond to EPC induction protocols. The current study further discovered increases in ANG1 and NRP1 gene expression in differentiated EPCs that may contribute to angiogenesis or repair of injured tissue.

Implantation of NSCs to replace, support, or guide nerve regeneration has potential use in both the CNS and peripheral nervous system (PNS). NPCs are thought to possess the capability of self-renewal and clonal isolation with great potential to generate neuronal^27^, glial^28^, and oligodendrocyte cells^29^ in the presence of a neurotrophic cocktail, such as bFGF, epidermal growth factor, and brain-derived neurotrophic factor (BDNF). Neuron-like cells, with positive immunostaining for Tuj1, MAP2, NeuN, and synapsin, can be induced by treating ASCs with a neuron induction cocktail medium^30^. Stimulation of neurotrophic and angiogenic properties in ASCs was achieved by *in vitro* incubation of the cells in neurotrophic medium followed by incubation in a medium with angiogenic chemicals^31^. The stimulated ASCs showed increased gene expression of BDNF, glial cell-derived neurotrophic factor, VEGF-A, and ANG1 proteins. The formation of neurospheres was established by simply seeding the human ASCs on chitosan-coated surfaces without various types of neurotrophic factors^21^. Although the fabrication of chitosan into neural tubes showed improvement in bridging nerve regeneration after spinal cord transection^32^ and peripheral nerve gap^22^, the current study only used chitosan-coated surfaces as an induction microenvironment to facilitate ASCs to form neurospheres. The neurospheres were positive for nestin, NF-H, and GFAP within the sphere (Fig. 1), and differentiated into neurons or astrocytes after transplantation into the HI-injured rats (Fig. 4). Hence, the current study demonstrated the therapeutic potential of autologous stem cells by offering an alternative source for EPCs and NPCs from ASCs.

After HI brain injury, the pathogenic responses of cerebrovascular structures and how they contribute to perinatal brain damage are not fully understood. Chemokines are released from cells in injured tissue to modulate several crucial processes, such as activation and recruitment of inflammatory cells, dissociation of endothelial tight junctions, and triggering or prevention of neuronal death. Some molecular mechanisms after HI injury include the activation of hypoxia-inducible transcription factors for regulation of erythropoietin for cell survival and VEGF to activate endothelial cells for capillary sprouting^33^. On the other hand, prenatal hypoxia causes fetal brain injury and is associated with HI-induced inflammation to induce the pro-apoptotic proteins Bax, Bcl-2, and p53 as well as pro-inflammatory cytokines^34^. Maternal hypoxia has been shown to increase the serum protein levels of interleukin-6 and tumor necrosis factor alpha in fetal guinea pigs^35^. Stem cells can release anti-inflammatory and pro-survival factors to benefit injured tissue via direct cell-cell interactions and paracrine factors. Significant protection from hippocampal and cortical volume loss was achieved by administering concentrated conditioned medium from cultured rat ASCs through the jugular vein to neonatal rats 1 hr before or 24 hrs after HI injury^19^. Insulin-like growth factor-1 and BDNF were identified as potential neurotrophic factors in ASC conditioned medium. In the current study, changes in endothelial and neuronal markers were observed after differentiating the ASCs into EPCs and NPCs (Fig. 6b). The profile of secretory factors may change in these differentiated progenitor cells and may require specific analysis to identify the potential cytokines in each specific progenitor cell and E+N combination in future studies.

The combination of E+N showed comprehensive therapeutic effects in the neonatal HI brain injury (Table 1). However, a combination with other cells may also benefit the EPCs or NPCs effect. Since the human ASCs also protected the traumatic brain injury in rats^36^, we further tested the three combination of ASCs, EPCs, and NPCs in our injury model. The same total amount of transplanted cells (2 × 10^5^ cells with equal one-third of ASCs, EPCs, and NPCs) was injected as aforementioned to compare the difference of treatment with ASCs only or the E+N combination (Fig. S7). However, the triple combination (A+E+N) did not exhibit an additional benefit compared to the E+N combination in ability; to reduce the brain damage score (Fig. S7A), to preserve the number of live neurons in Nissl staining (Fig. S7B), or to prevent cell death via TUNEL staining (Fig. S7C). We also tested the cell motility of the triple combination under normal and hypoxic conditions in Boyden chambers (Fig. S7D). The same amount of cells in triple combination did not show further synergistic enhancement in mobility when compared to the E+N combination (Fig. S7E). One possibility might be due to the adversarial effect of adding ASCs into the triple combination resulting in the reduction of cell number for EPCs and NPCs, a dilution effect. Different ratio of cell combinations might need to be considered and optimized in future studies.

The niche (or microenvironment) in ischemic tissue may promote the mobilization and recruitment of stem/progenitor cells to the injured site to improve tissue repair. Members of the chemokine family are divided into four groups depending on the spacing of their first two cysteine residues. Among different chemokines, stromal cell-derived factor-1 (SDF-1 or CXCL12) is a major chemokine that stimulates chemoattraction of hematopoietic stem cells and EPCs. SDF-1 also plays an important role in multiple processes after ischemic stroke, including the inflammatory response, focal angiogenesis, and the recruitment of NPCs to the injured site^37^. The engraftment and neo-angiogenesis by trafficking of EPCs from peripheral blood participates in vasculogenesis in animals with hind limb ischemic injury^38^. Intravenous transplantation of human ASCs protects the rat brain from traumatic brain injury^36^. The *in vivo* near-infrared IVIS imaging of DiR-labeled ASCs demonstrated the bio-distribution of transplanted ASCs in brain, spleen, lung, and liver. The functional improvement of HI injury maybe due to the protection and/or repair of the microcirculation in the vascular structure of the brain. Prevention of cell apoptosis (Fig. 2d) and engraftment of transplanted cells (Fig. 4a) were observed with the combination of EPCs and NPCs. In addition, the E+N combination facilitated neurogenesis (Fig. 4b) and angiogenesis (Fig. 4c). Although specific antibodies for humans and rats may be useful for distinguishing endogenous cell repair and regeneration, the contributions of the host and donor cells to protecting and/or repairing the neurovascular structure following transplantation of EPCs and NPCs require further labeling of specific cells, such as perhaps GFP to label EPCs and red fluorescent protein to label NPCs. Tracing of fluorescent-labeled cells in neurovascular structures after specific inhibitor treatments of EPCs and NPCs will allow dissection of the complex relationships between EPC-NPC and host-donor interactions in future studies.

## Conclusion

The current study demonstrates that the combination of EPCs and NPCs shows a promising outcome in protecting neonatal brain from injury. ASCs can be differentiated into specific EPCs and NPCs. The best therapeutic effects of the combined therapy were confirmed in terms of protection from brain tissue loss and cell apoptosis, preservation of neurovascular structures, and improvements in functional recovery. Transplantation of single types of EPCs or NPCs suggests that different specified cells have distinct therapeutic effects in neurovascular structures and elicit particular functional consequences. Cell migration was promoted by cell-cell interactions between EPCs and NPCs, which were further enhanced in hypoxic microenvironments. These results suggest that the NPCs and EPCs derived from ASCs are a potential cell source for cell therapy in HI brain injury.

## Methods

### Isolation of ASCs

Human ASCs from healthy donors were obtained via liposuction with informed consent to protect client information and patient rights as approved by the Institutional Review Board (IRB) of the National Cheng Kung University Hospital (NCKUH). The methods for isolation and usage of human ASCs in current study were carried out in accordance with the IRB approved guidelines. To assure the therapeutic potential of ASCs, ASCs were also isolated from Sprague-Dawley rats provided by the animal center with the approval of the Institutional Animal Care and Use Committee at NCKU. The ASC isolation protocols were established by Dr. Patricia Zuk and Dr. Marc Hedrick at UCLA^17^. The culture of ASCs and assessment of multi-lineage differentiation capability were performed according to the previous study^21^.

### Induction of endothelial and neural differentiation

Endothelial differentiation of ASCs was induced by the combination of chemical growth factors and mechanical shear stress as previously described^23^. For endothelial differentiation, the ASCs were pre-treated with EGM (Lonza) for 3 days and then treated with LSS for 24 hrs. The LSS system was created by sandwiching a silicon gasket between an acrylic base and a cell-seeded glass slide according to our previous study^39^. To test the differentiation of EPCs from ASCs, the PCR was used to evaluate endothelial gene expression, including expression of VEGF, Flt-1, Flk-1, vWF, and PECAM-1. The detailed primer sequences, cycle conditions, and annealing temperature of the primers were reported in a previous study^23^. Immunofluorescent staining was performed to confirm the endothelial protein expression of vWF (1:200, Santa Cruz Biotechnology) and PECAM-1 (1:200, Abcam). To test the endothelial function in differentiated cells *in vitro*, LDL uptake and tube-like structure formation on Matrigel were assessed as previously described^23^.

The differentiation of NPCs from ASCs on chitosan-coated surfaces was described in a previously paper^21^. For neural sphere formation, 1% w/v chitosan (Sigma) was applied to regular tissue culture plates and neutralized with 1 N NaOH solution. The differentiation conditions, seeding density (2 × 10^4^ cells/cm^2^), and harvesting time (72 hrs) were determined by optimal sphere formation with high cell viability on a chitosan-coated surface. Neural lineage markers were assessed with nestin, NF-H, and GFAP using PCR for measuring gene expression and immunofluorescent staining to confirm protein expression. Gene expression in differentiated cells was normalized using the endogenous reference gene GAPDH and the ratios to undifferentiated ASCs to provide fold changes of target gene expression levels.

### HI animal model and cell-based therapies

To evaluate the therapeutic effect of different cell types, we used the HI encephalopathy rat model as described in previously^24^. The Sprague-Dawley rats were provided by the animal center with the approval of the animal use protocol by the Institutional Animal Care and Use Committee (IACUC) at NCKU. All experimental protocols were approved by the IACUC at NCKU and the experimental methods were carried out in accordance with the approved guideline. On postnatal day 7, the rat pups were divided into five groups and given PBS injection or transplantation of ASCs, EPCs, NPCs, or a combination of EPCs and NPCs (E+N). The HI injury model was created by anesthetizing the rat pups with 2.5% halothane and then permanently ligating the right common carotid artery with 5-0 surgical silk. After ligation for 1 hr, the rat pups were placed in a 37°C water bath and 8% oxygen for 2 hrs to create the hypoxic injury after brain ischemia. Different specified cells (2 × 10^5^ cells) were intraperitoneally transplanted into the HI-injured rat pups. To avoid leakage and cell loss during injection, the transplantation was divided into two parts, immediately before and after the hypoxic treatment. At least three repetitions of each treatment were performed for every evaluation, including TTC, brain tissue loss and cell apoptosis, NVU structures, and functional assessments.

### Assessments of Brain Damage

The short-term protection provided by the cell therapy was investigated by harvesting brain tissue samples after receiving various cell transplantations for 7 days (on postnatal day 14). TTC, Nissl, and TUNEL staining were performed to evaluate the brain damage after HI injury and the preservation with different cell therapies. To determine the severity of brain damage, TTC staining was used to measure the infarct volume by staining the viable tissue red with the TTC metabolite and leaving the dead tissue unstained^40^. For Nissl and TUNEL staining, the rat brains were perfused with saline, fixed with 4% paraformaldehyde at 4°C overnight, cryoprotected in 30% (w/v) sucrose in 0.1 M PBS, and then serially and coronally sectioned into 20-μm thick slices from the genu of the corpus callosum to the end of the dorsal hippocampus. The cross-sectional areas of the striatum, cortex, and hippocampus were obtained from the five reference planes approximately corresponding to plates 15, 18, 27, 31, and 39 in the rat brain atlas^41^. After staining with 0.1% cresyl violet solution for 1 hr, neurons were identified by cresyl violet-stained Nissl bodies, and the area was measured manually using computerized image analysis software (Image-Pro Plus 4.5). The percentage of average brain area lost was calculated by subtracting the intact brain area (left brain) from the injured brain area (right brain) and then dividing by the intact brain area. The prevention of cell apoptosis after transplanting various specified cells was examined with an *in situ* TUNEL assay according to the recommended protocols of a commercial apoptosis kit (Roche). Briefly, detection of DNA strand breaks in individual cells was achieved by incubating the brain sections with the TUNEL reaction mixture containing TdT and fluorescein dUTP. The incorporated fluorescein was detected by conjugating an anti-fluorescein antibody and then visualizing the immunocomplex with a POD substrate reaction. The percentage of apoptotic cells was quantified using HistoQuest software (TissueGnostics Co.).

### Immunofluorescent staining of NVU structures

The cross-sectional brain samples were stained with endothelium markers using specific antibodies against RECA (1:200, Abcam) or PECAM-1 (1:100, Upstate) to visualize the vascular structures. The IB4 antibody (1:200, Invitrogen) was used to indicate angiogenesis. Vessel density was measured by positive RECA staining in each captured image and was normalized to brain samples without cell therapy (PBS injection). The neural structures in HI-injured brains were recognized by various antibodies against NeuN (1:200, Abcam) for the nuclei of neurons, GFAP (1:200, Abcam) for astrocytes, and nestin (1:200, Abcam) for NSCs. Fluorescent secondary antibodies (1:200, Invitrogen) were used to specifically label the primary antibodies for illustrating the NVU structures. The tissue integrity of neurons and vascular structures among different brain areas was visualized with a tissue scanning fluorescent microscope (BX51, Olympus) with a 20 × objective lens. At least three random images were selected to calculate the number of NeuN (+) cells per visual field for neuron numbers. The percentage of normal astrocytes was calculated by dividing the number of astrocytes with reactive phenotype by the total number of GFAP (+) cells.

### Cell tracing

To investigate progenitor cell homing and engraftment into the HI-injured brain for structural restoration, the GFP gene was delivered into different cell types using an adenovirus. In the brain tissue studied for long-term effects (harvested after 1 month of transplantation), the GFP label may be degraded after injecting fluorescent-labeled cells into the complex environment of the injured rats. Therefore, a specific antibody against hChromatin (1:200, Merck Millipore) was used to identify these injected human cells. The engraftment of donor cells into the neurovascular structures was demonstrated by double staining of GFP with the specific markers for vessels, astrocytes, and neurons.

### Functional assessments

After the injured rat pups received therapy for 1 month, cognitive and motor tests were performed to evaluate long-term functional recovery. For learning and memory tests, the rats were placed in the Morris water maze that had a platform and landmarks of the platform between postnatal day 21 and 24 with four training trials per day. The latency time for rats to reach the platform and the total distance traveled were recorded in different trials. On postnatal day 25, the rats were placed into the same pool without a platform, and memory function was evaluated by recording the frequency at which rat pups crossed the previous platform location. Motor function was assessed by grip strength tests on postnatal day 21 and 25. The maximum grip forces were measured by placing the forelimb of the rats on a T-bar attached to a load cell to record the maximum force when pulling them off from the bar. At least three trials were performed, and the maximum force was averaged on each measuring day per rat. More than three rats were used for each therapeutic group in the cognitive and motor functional tests. The brain samples were harvested immediately after the measurement of motor function on postnatal day 25.

### Transmigration Assay

The complex microenvironments in the injured brain of HI rats may trigger the motility of transplanted cells. The chemotaxis and trans-migration ability were measured using a Boyden chamber assay by loading specific types of cells (2 × 10^4^ per well) into 8 μm-pore membranes (Neuro Probe) and treating them with or without the hypoxia mimetic reagent DFO (50 μM, Sigma) to mimic *in vivo* hypoxic conditions. After disassembling the Boyden chamber, the transmigrated cells were fixed and stained with Giemsa for 15 min to quantify cell numbers. To illustrate the possible mechanism for the cell-cell interaction between EPCs and NPCs, the NRP1 blocking peptide (DG2, 20 μM, Digitalgene), the SDF-1/CXCR4 pathway inhibitor (AMD3100, 8 μg/mL, Sigma), and the FGFR1 inhibitor (SU5402, 20 μM, Sigma) were applied to EPCs and NPCs separately for 1hr. The treated cells (inhibitor or vehicle) were then rinsed with PBS, passaged with trypsin, centrifuged, and re-suspended in regular medium for loading into the Boyden chamber.

### Statistical analyses

The data are expressed as the mean ± standard deviation. Statistical analysis was performed using the one-way analysis of variance (ANOVA) with the Scheffé's post-hoc test. Values of p < 0.05 were considered statistically significant. All statistical analyses were performed using graphing and analysis software (Origin version 8.5, OriginLab).

## Additional Information

**How to cite this article**: Hsueh, Y.-Y. *et al*. Synergy of endothelial and neural progenitor cells from adipose-derived stem cells to preserve neurovascular structures in rat hypoxic-ischemic brain injury. *Sci. Rep*. **5**, 14985; doi: 10.1038/srep14985 (2015).

## Supplementary Material

Supplementary Information

## Figures and Tables

**Table 1:** Summary of therapeutic effects among different types of cell-based therapy.

Therapeutics effect	Treatments				
	PBS	ASC	EPC	NPC	E+N
Prevent infraction	—	+	+ +	+ +	+ +
Alive neuron area	—	+	+	+	+ +
Prevent cell death	—	+	+ +	+ +	+ +
Vessel structure	—	+	+ +	+	+ +
NeuN neuron	—	+	+ +	+ +	+ +
Normal astrocyte	—	+	—	+	+
Memory function	—	—	—	—	+
Motor function	—	+	+	—	+

ASC, adipose-derived stem cell; EPC, endothelial progenitor cell; NPC, neural progenitor cell; E+N, combination of EPC and NPC.
