# Corrigendum: Synergy of endothelial and neural progenitor cells from adipose-derived stem cells to preserve neurovascular structures in rat hypoxic-ischemic brain injury

**DOI:** 10.1038/srep31255

**Published:** 2016-11-14

**Authors:** Yuan-Yu Hsueh, Ya-Ju Chang, Chia-Wei Huang, Fitri Handayani, Yi-Lun Chiang, Shih-Chen Fan, Chien-Jung Ho, Yu-Min Kuo, Shang-Hsun Yang, Yuh-Ling Chen, Sheng-Che Lin, Chao-Ching Huang, Chia-Ching Wu

Scientific Reports 5: Article number: 1498510.1038/srep14985; published online: 10 08 2015; updated: 11 14 2016

The original version of this Article contained errors in the affiliations.

‘Department of Cell Biology and Anatomy, National Cheng Kung University, North District, Tainan City, Taiwan’

was incomplete, and now reads:

‘Department of Cell Biology and Anatomy, College of Medicine, National Cheng Kung University, Tainan, Taiwan, ROC’

This error has now been corrected in the HTML and PDF versions of this Article.

