# Supplementary material for: Synergy of endothelial and neural progenitor cells from adipose-derived stem cells to preserve neurovascular structures in rat hypoxic-ischemic brain injury
Source: Sci Rep. 2015 Oct 8;5:14985. doi: 10.1038/srep14985 (PMC4597209; doi:10.1038/srep14985)
Supplement: Supplementary Information [file srep14985-s1.doc]

**Synergy of endothelial and neural progenitor cells from adipose-derived stem cells to preserve neurovascular structures in rat hypoxic-ischemic brain injury**

Yuan-Yu Hsueh^1,2§^, Ya-Ju Chang^3,4§^, Chia-Wei Huang^4§^, Fitri Handayani^3^, Yi-Lun Chiang^3,4^, Shih-Chen Fan^5^, Chien-Jung Ho^2,7^, Yu-Min Kuo^3,4^, Shang-Hsun Yang^4,6^, Yuh-Ling Chen^8^, Sheng-Che Lin^1^, Chao-Ching Huang^2,7, 9^, Chia-Ching Wu^3,4,10, 11*^

^1^ Division of Plastic Surgery, National Cheng Kung University Hospital

^2^ Institute of Clinical Medicine, National Cheng Kung University

^3^ Department of Cell Biology and Anatomy, National Cheng Kung University

^4^ Institute of Basic Medical Sciences, National Cheng Kung University

^5^ Department of Occupational Therapy, I-Shou University

^6^ Department of Physiology, National Cheng Kung University

^7^ Department of Pediatrics, Taipei Medical University

^8^ Institute of Oral Medicine, National Cheng Kung University

^9^ Department of Pediatrics, Wan-fan Hospital, College of Medicine, Taipei Medical University

^10^ Department of Biomedical Engineering, National Cheng Kung University

^11^ Medical Device Innovation Center, National Cheng Kung University

Corresponding address:

Chia-Ching Wu (joshccwu@mail.ncku.edu.tw)

No. 1, University Rd., Tainan, Taiwan

Tel: +886-6-2353535 ext 5327, Fax: 886-6-2093007

^§^ Co-first author

^*^ Corresponding author


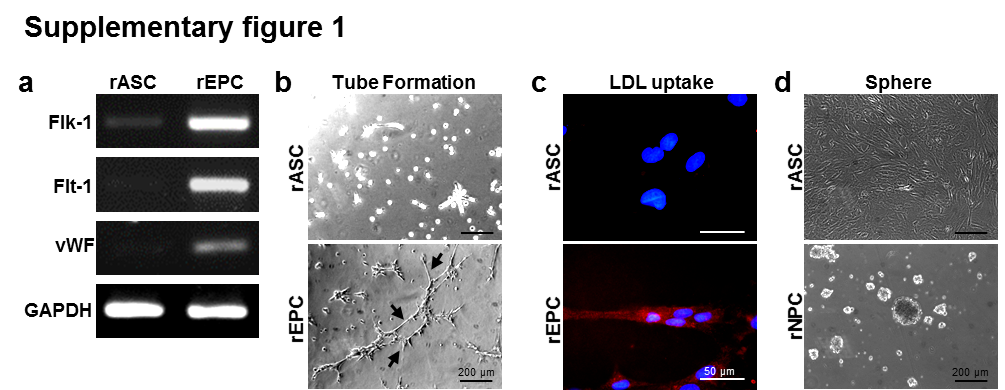


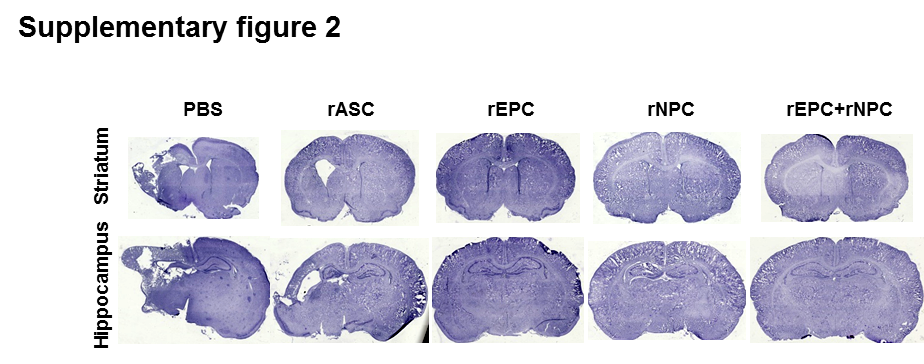


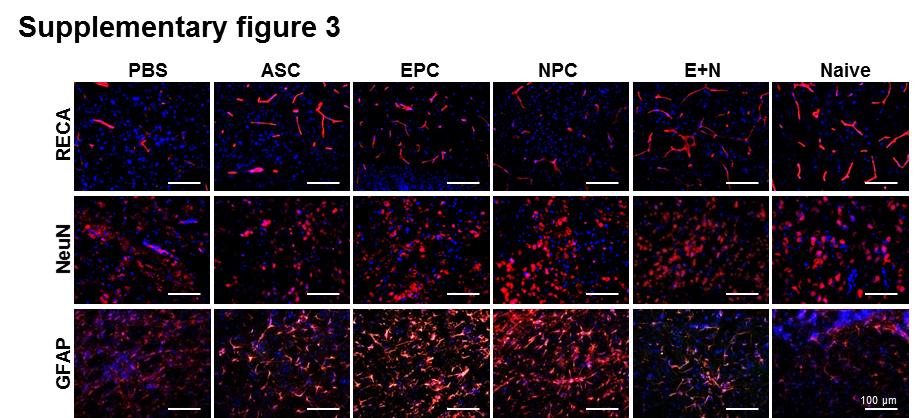


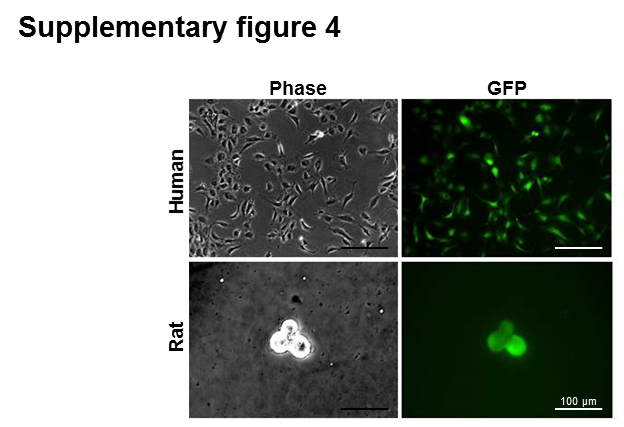


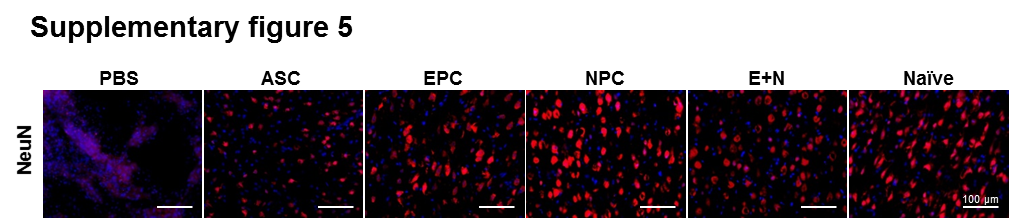


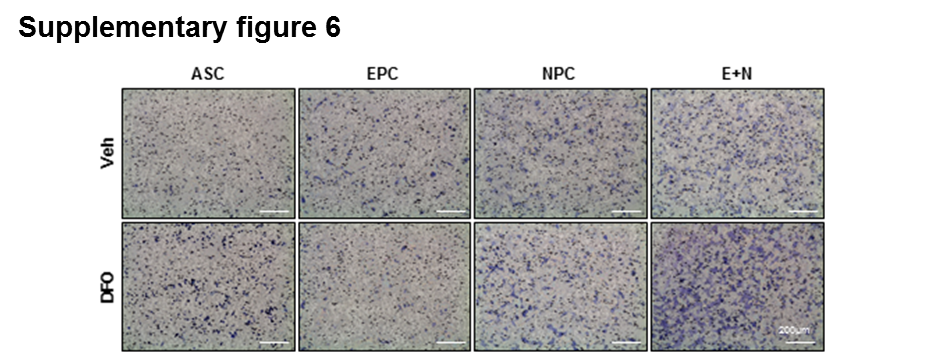


**
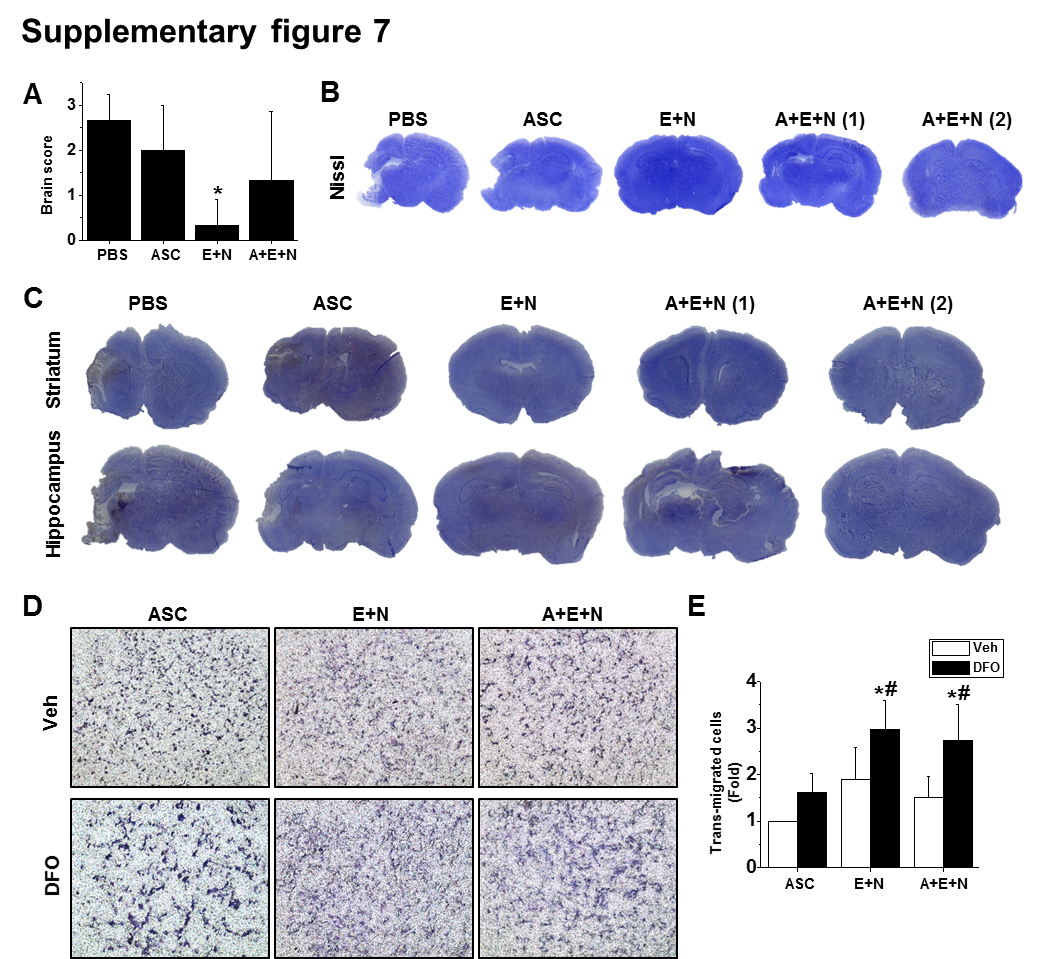
**

**Supplemental Figure legends**

Supplementary figure 1. The EPC induction methods also increased Flk-1, Flt-1, and VE-Cadherin (VE-Cad) gene expression in ASCs isolated from Sprague-Dawley rats (rASCs) (n=3) (A). The differentiated rat EPCs (rEPCs) showed tube-like structures on Matrigel (n=3) (B) and uptake of DiI-labeled LDL (n=3) (C), demonstrating endothelial functions. The rASCs also formed sphere-like structures when seeded on chitosan-coated surfaces for induction of NPCs (rNPCs) (n=3) (D).

Supplementary figure 2. The specified cells derived from rASCs also showed a similar ability to protect the brain from HI injury as demonstrated with Nissl staining (n=3).

Supplementary figure 3. The protection of neurovascular structures by various cell therapies was also assessed in the striatum of the injured hemisphere using specific antibodies against RECA (n=4), NeuN (n=4), and GFAP (n=4).

Supplementary figure 4. High transduction efficiency was established with an adenovirus to label the human and rat cells with GFP before cell transplantation. The induction of specified cells, such as neurosphere formation in rASCs (lower panel), did not alter the GFP expression (n=3).

Supplementary figure 5. Neurovascular structures in rats after receiving various cell therapies for 18 days (postnatal day 25). The brain samples were harvested immediately after the motor function assessments and immunostained with NeuN for neurons (n=3) and RECA for vessel structures (n=3).

Supplementary figure 6. The combination of EPCs and NPCs from rat ASCs also increased transmigration in a Boyden chamber under normoxic conditions, and the application of the hypoxia mimetic reagent DFO also promoted the increase of mobility in rat cells (n=5).

Supplementary figure 7. Triple combination of ASCs, EPCs, and NPCs (A+E+N) did not further improve the therapeutic outcome and E+N combination in reducing the brain damage score (n=3) (A), preserving the living neurons in Nissl staining (n=3) (B), nor preventing the cell death in TUNEL staining (n=3) (C). *p < 0.05 compared to the injection of PBS. The Boyden chamber assay also demonstrated the similar cell mobility of triple combination as comparing to the combination of E+N both under normal and hypoxic condition (n=3) (D). The addition of ASCs into EPCs and NPCs (A+E+N) enhanced cell migration than ASCs only, but did not show significant difference as compare to E+N combination (n=3) (E). *p < 0.05 compared to undifferentiated ASCs under normoxia. #p < 0.05 compared to undifferentiated ASCs under hypoxia.
